# The other-race effect and holistic processing across racial groups

**DOI:** 10.1038/s41598-021-87933-1

**Published:** 2021-04-19

**Authors:** Hoo Keat Wong, Alejandro J. Estudillo, Ian D. Stephen, David R. T. Keeble

**Affiliations:** 1 School of Psychology, University of Nottingham Malaysia, Semenyih, Malaysia; 2 Department of Psychology, Bournemouth University, Dorset, UK; 3 Department of Psychology, Macquarie University, Macquarie Park, Australia; 4 Perception in Action Research Centre, Macquarie University, Macquarie Park, Australia

**Keywords:** Psychology, Human behaviour

## Abstract

It is widely accepted that holistic processing is important for face perception. However, it remains unclear whether the other-race effect (ORE) (i.e. superior recognition for own-race faces) arises from reduced holistic processing of other-race faces. To address this issue, we adopted a cross-cultural design where Malaysian Chinese, African, European Caucasian and Australian Caucasian participants performed four different tasks: (1) yes–no face recognition, (2) composite, (3) whole-part and (4) global–local tasks. Each face task was completed with unfamiliar own- and other-race faces. Results showed a pronounced ORE in the face recognition task. Both composite-face and whole-part effects were found; however, these holistic effects did not appear to be stronger for other-race faces than for own-race faces. In the global–local task, Malaysian Chinese and African participants demonstrated a stronger global processing bias compared to both European- and Australian-Caucasian participants. Importantly, we found little or no cross-task correlation between any of the holistic processing measures and face recognition ability. Overall, our findings cast doubt on the prevailing account that the ORE in face recognition is due to reduced holistic processing in other-race faces. Further studies should adopt an interactionist approach taking into account cultural, motivational, and socio-cognitive factors.

## Introduction

The other-race effect (ORE; also known as the own-race bias) is a well-documented phenomenon showing that people are generally better at recognizing faces of their own race, compared to faces of different races. It exists across different countries and ethnic groups^1^ and is evident not only in laboratory settings but also in real-world scenarios^2^. Although the ORE has been extensively studied for the last four decades, the specific mechanisms underlying this effect are still poorly understood. The present paper aims to shed light on this issue by exploring the holistic processing account of the ORE^3^.

According to a long-standing scientific tradition, holistic processing is the hallmark of adults’ expert face recognition^4^. While the exact definition of holistic processing is a matter of ongoing debate, it is widely accepted that when adults perceive faces holistically, the facial components (e.g., eyes, nose, mouth) are integrated into a whole or gestalt-like representation^4,5^. Two experimental paradigms have been widely employed as standard measures of face-specific holistic processing: the whole-part task and the composite face task. In the whole-part task^6,7^, recognition memory of a facial part (e.g., the eyes) is more accurate when it is presented in the context of a whole face than in isolation, suggesting that facial features are embedded into a holistic face percept. In the composite face task^4^, observers’ performance on matching two identical top face halves is better when these top halves are misaligned (i.e., spatially offset) with different bottom halves than when the top and the bottom parts are aligned. This composite effect demonstrates that the face parts are not perceived independently from the whole face.

Holistic processing has been proposed as one important mechanism underlying the ORE. According to this view, in contrast to own-race faces, people are inefficient at integrating facial components from other races into a whole representation^8,9^, and therefore other-race faces might be subject to weaker holistic processing than own-race faces. Although a stronger holistic processing for own-race faces compared to other race faces has been reported using the whole-part task^9^ and the composite face task^10^, these results are not always replicated^11–13^. In fact, the results obtained from the composite task are very inconsistent^8,11,14^, and certainly not as consistent as those from the whole-part task. The discrepancy in the holistic effect results may stem from methodological differences between studies (e.g., face size^15^, measuring methods^10,16^, limited construct validity of holistic processing^17–19^, and independent sample collection from race groups who have differential level of interracial experience^9,10,12^). Yet, these observations lend support to the claim that the holistic mode of processing faces allows efficient encoding of an individual face^20^ and can be moderated by the race of observer^21^.

Limited experience with other-race faces has been proposed as one of the causes of the reduced holistic processing for other-race faces, and therefore the robust ORE. For example, in the aforementioned studies, Caucasian observers had very limited exposure to Asian faces in either daily life or the media; in contrast, Asian participants in the these studies were international students in Western universities and reported having similar amount of social contact with own-race and other-race individuals^8,22^. Yet, this experience-based explanation of holistic processing has been questioned because other studies have found equivalent levels of holistic processing for both own- and other-race faces in Asian participants with limited exposure to other-race faces^10,12,13,23,24^.

An explanation for the roughly equivalent holistic processing magnitude for own-race and other-race faces found in the Asian samples is that, compared to Caucasians, Asians are more prone to holistic processing of both face and non-face visual stimuli. For example, Asian observers exhibit a stronger global processing bias in the classical Navon task than Caucasian observers^25^. Not only does this theoretical explanation underline the cultural differences in cognitive styles between Caucasians and Asians, but it also implies that holistic processing detected for other-race faces in Asian participants may be attributable to domain-general global processing bias instead of specialised higher-level mechanisms for face recognition, as argued by Michel et al.^10,26^. Based on such a general cognitive style, Asians may maintain a relatively broad facial representation that is advantageous for recognising both own- and other-race faces, thereby reducing the ORE. This may further explain why some researchers failed to observe the ORE in Asian samples^27,28^. Although empirical studies have set out to explore the association between domain-general global processes, face recognition ability, and face-specific holistic processing^29,30^, only a few studies directly evaluated its validity by comparing between multiple ethnic groups with the use of face stimuli of different races. For instance, DeGutis et al.’s^16^ and Wang et al.’s^31^ conclusion that recognition ability is strongly linked to the magnitude of holistic processing lack external validity as the former study only tested a Caucasian participant sample with the use of Caucasian faces, whereas the latter study did not report the race of participants and only used Asian face stimuli.

### The present study

The widespread assumption in the face perception literature is that the whole-part and the composite face tasks measure the same underlying (holistic) mechanisms^32–36^. However, a recent study found no association between these two tasks^37^, suggesting that they, in fact, tap different perceptual mechanisms. So far, only one recent study^13^ employed both composite-face and whole-part tasks to index holistic processing while comparing between two different race groups (Caucasian vs. Chinese). Mondloch et al. reported evidence that the magnitude of holistic processing for own-race and other-race faces did not differ in both Caucasian and Chinese adults. However, this cross-racial study did not measure participants’ face recognition memory and therefore it remains unclear to what extent holistic processing affects the ORE in recognition memory.

In the present study, we investigate whether the ORE in face memory can be attributed to reduced holistic processing (as indexed by both composite-face and whole-part effects) of unfamiliar other-race faces. To increase the generalizability of our results, we test face recognition ability and holistic processing in Malaysian Chinese, African, European Caucasian, and Australian Caucasian young adults using three races of faces (Chinese, Caucasian and African faces). If holistic processing is important for recognising faces and individual-level face discrimination experience is crucial for holistic processing to develop, we would expect that participants from different race groups will show the typical ORE in face memory, and stronger holistic processing for own-race faces than other-race faces. Alternatively, if holistic processing can be generalised to facial morphologies that are less visually experienced without extensive individuating (e.g.^38–40^), both own- and other-race faces would elicit holistic effects of similar magnitudes across race groups.

In addition, we used Navon figures to compare global–local processing differences between the four race groups. Based on the accumulated evidence of stronger global processing but weaker local processing in East Asians compared to Western Caucasians^41^, we predicted that Malaysian Chinese would be more susceptible to global–local interference (GLI)—an index of the tendency to globally process general objects—than Caucasian groups (European and Australian). Such a perceptual difference indicates that information-gathering strategy (global versus local processing) for general stimuli can be culture-dependent^25,42^, with collectivist societies (i.e., the East) producing a preference for integrating context, and individualist societies (i.e., the West) producing a preference for ignoring context^43^. Like South-East Asia, African cultures are also considered collectivistic^44^, but research on cultural differences in perceptual processing bias has often neglected this population. To ensure valid theoretical conclusions, we also tested African participants from collectivistic societies and hypothesised that they would show an evident GLI (i.e. faster and more accurate at global processing).

Furthermore, if the mechanisms involved in holistic processing can apply to other object classes (e.g. Navon letters) and are not specialised for faces per se (“domain-generality hypothesis”), then GLI scores would vary systematically with performance on both the whole-part task and the composite face task. Conversely, if special mechanisms are involved in processing faces holistically (“domain specificity hypothesis”), the magnitude of GLI would not correlate with holistic face processing measures and face recognition ability, such that perceptual biases for general information processing is not necessarily generalisable to high-level, specialised face processing.

## Method

### Participants

Thirty-one Malaysian-Chinese (16 females; *M*_*age*_ = 21.65, *SD* = 2.6), 30 European Caucasians (14 females; *M*_*age*_ = 22.40, *SD* = 3.10), 30 Australian Caucasians (23 females; *M*_age_ = 21.03, *SD* = 4.45), and 30 Africans (12 females; *M*_age_ = 26, *SD* = 5.5) took part in this study. All participants self-reported single rather than mixed-race descent. Malaysian Chinese were students studying at the University of Nottingham Malaysia. They were all born and grew up in Malaysia. None of them reported spending more than 9 months outside Malaysia. European Caucasian and African participants were international students recruited at the University of Nottingham Malaysia. European-Caucasians were mostly British (one Italian, one Dutch) who had resided in Malaysia for 6.5 months on average. None reported spending more than 2 years in a predominantly Asian country. African participants were mostly Nigerians (five Kenyans, two Zimbabweans, one Zambian, one Somali) who had resided in Malaysia for 1.5 years on average. Australian-Caucasian participants were recruited from Macquarie University, Sydney. All were born in Australia and had not lived in a predominantly Asian country for more than 4 months (*M* = 5.4 days, *SD* = 21, range 0–120 days). All participants reported having normal or corrected-to-normal vision and having no difficulty with face recognition. All experimental protocols were approved by the University of Nottingham Malaysia, Faculty of Science Ethics Committee, and all methods were carried out in accordance with guidelines of the British Psychological Society. The individuals depicted in all figures signed a written informed consent for their images to be published. Participants gave written informed consent prior to the experiment and received either course credit or monetary compensation of RM10 (approximately US$3) for their participation.

A priori power analysis using G*Power 3.1.9.2^45^ showed that, for all of the terms in our analyses that directly related to our hypotheses (all of which are 4 × 3 within-between interactions in mixed ANOVAs), this sample size gave sufficient power to detect effect sizes of *η*_*p*_^2^ < 0.06 (a small-medium effect size), with α = 0.05, and power (1 − β) = 0.80.

### Apparatus, stimuli and procedure

**Figure 1:**
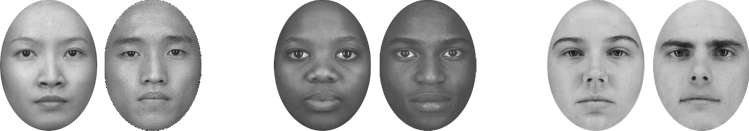
Examples of the three races of faces (i.e. Chinese, African and Caucasian faces) used in the face tasks. Each race pair shows a female (left) and a male (right) face. The individuals depicted in this figure signed a written informed consent to the publication of their facial images.

Chinese, Caucasian and African faces were used. Chinese facial images were collected from a student population at the University of Nottingham Malaysia Campus; Caucasian faces were obtained from students at Macquarie University, Australia. African faces were requested from Coetzee’s^46^ face database. All stimuli used in the face tasks were frontal images of young adult faces (both male and female) with neutral expression, and no glasses, facial hair, or distinctive blemishes (see Fig. 1). Individual face identities did not appear in more than one task. Considering that face photograph memorability is influenced by a combination of facial properties such as distinctiveness and attractiveness^47^, 216 face images (72 for each race) were originally sampled according to the results of a prior experiment in which each face race was matched in terms of attractiveness and distinctiveness as rated by 95 young adult participants (24 Chinese, 24 Malay, 25 Indian, and 21 Caucasian) on a 7-point Likert scale^48^. This selection criterion minimised potential confounds of facial distinctiveness and attractiveness on participants’ recognition performance. The original images were first cropped to form an ellipse shape that excluded external features (leaving a roughly oval shape with no hair on the top and sides). To minimise the low-level image cues (e.g., skin colour information), all face images were transformed into 8-bit grayscale images in Adobe Photoshop CS6 and were aligned on the eyes’ position using Psychomorph software^49^ (http://users.aber.ac.uk/bpt/jpsychomorph/, Version 6). Stimuli were presented on a 15.6″ monitor (resolution 1366 × 768). Participants were tested individually in a quiet dimly lit room with three face tasks (yes–no recognition task, composite task, and whole-part task), in counterbalanced order. Participants also performed a global–local task; however, as this task induces holistic or featural processing biases^50^, it was always performed last. Participants completed all tasks in approximately one hour, including breaks between each task.

#### Yes–no recognition task

**Figure 2:**
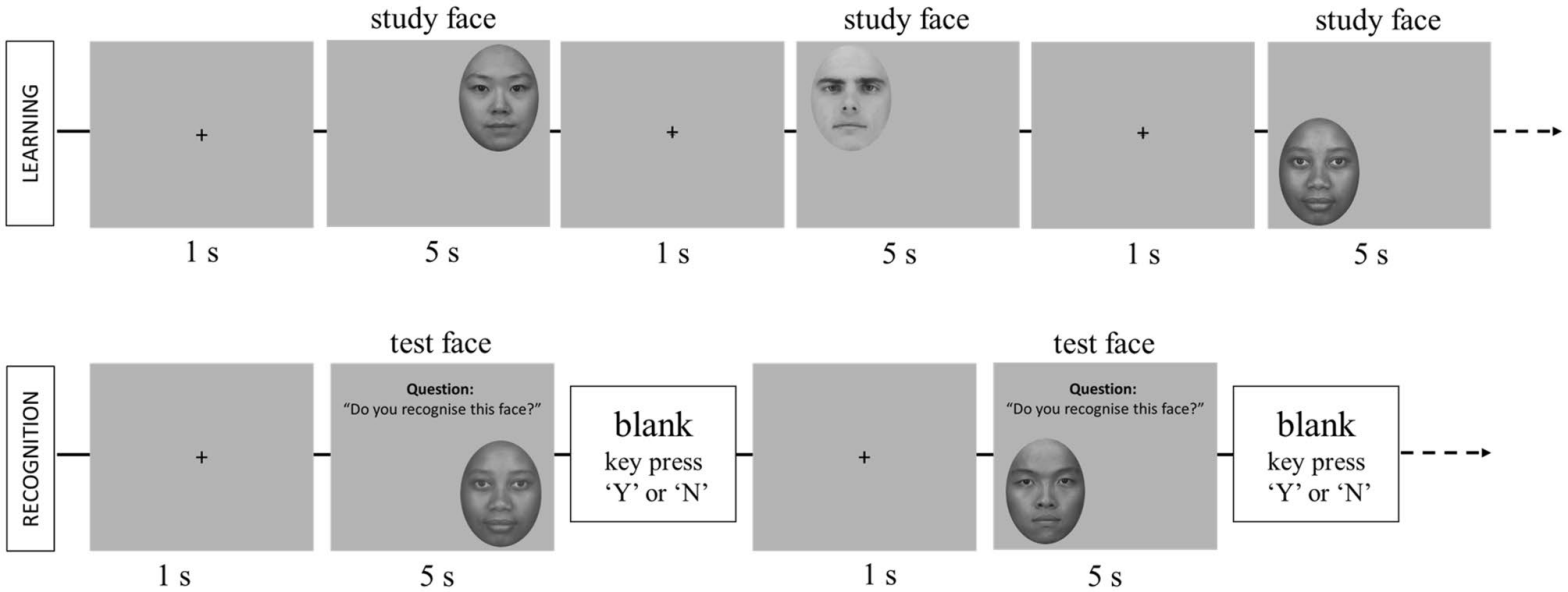
Experimental procedure for the learning and recognition stages in the yes–no recognition task.

Sixteen faces of each race group (eight females) were selected to form the experimental set. Each face was presented only once on a light grey background and sized 7.5° horizontal by 10.5° vertical at approximate viewing distance of 60 cm. During the learning phase, participants were asked to passively view and learn 24 faces (eight per race group). On each trial, a face was presented randomly in one of the four quadrants for 5 s, preceded by a central fixation cross for 1 s. In the recognition phase, 24 learned faces were randomly intermixed with 24 novel faces. For learned faces, the facial expression (neutral or smiling) changed between the learning and recognition phases to avoid a trivial image matching strategy. On each trial, participants were required to indicate as quickly and as accurately as possible whether they had seen the face in the learning phase. The face was presented for up to 5 s and no trial-by-trial feedback was given. If participants did not respond within the first 5 s, a blank screen would appear until they responded. Both response times and accuracy were recorded. Faces were presented in a random order, with the constraint that no more than three trials involving a given race occurred in immediate succession. The experimental procedure is illustrated in Fig. 2.

#### Whole-part task

**Figure 3:**
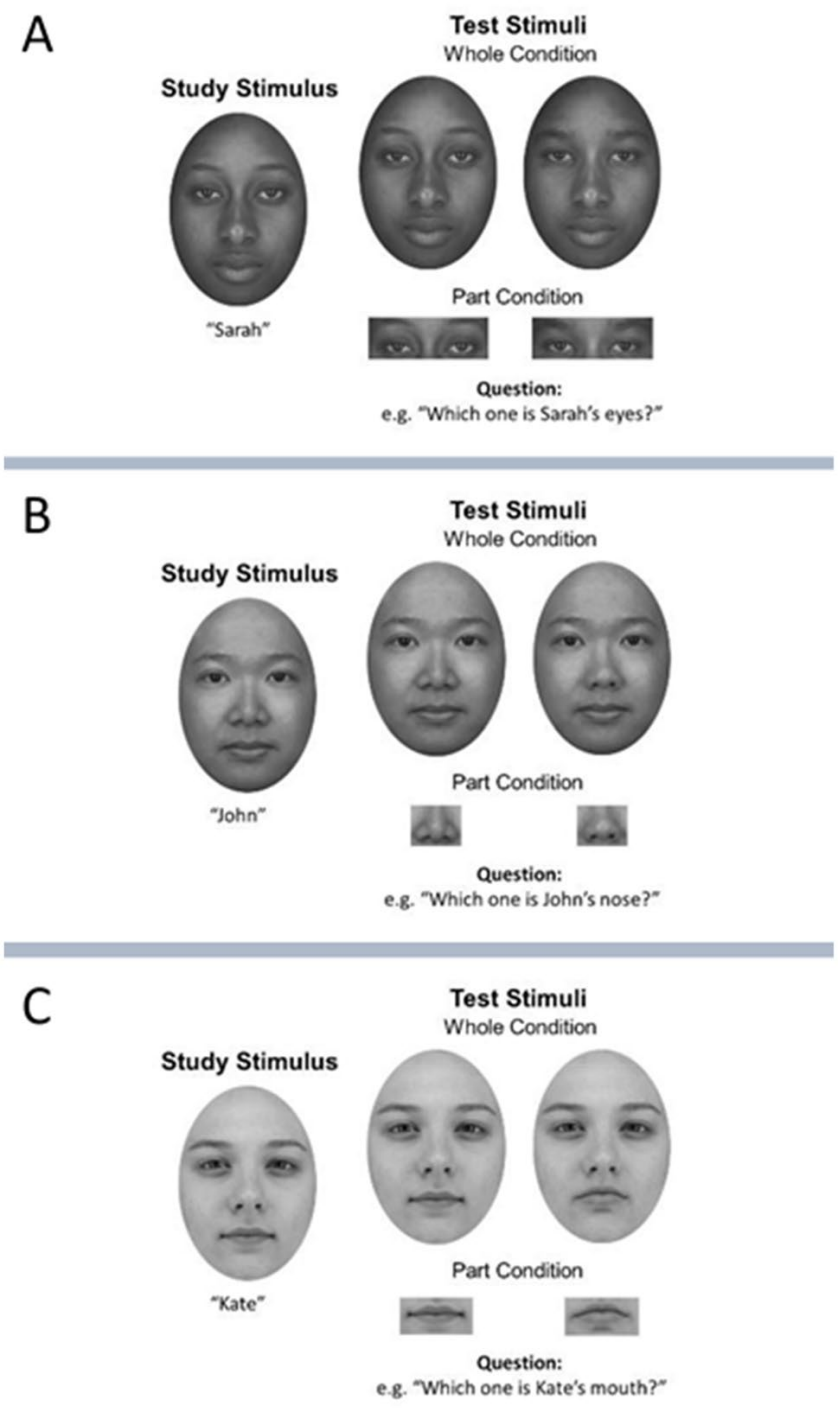
Example of the stimuli of three different races used in the whole-part task. The whole-part effect (*WPE*) relies on the assumption that it is much easier to identify the eyes (**A**), nose (**B**), or mouth (**C**) of the target face when the features are shown in the context of the whole face than when they are shown in isolation.

Stimuli were created from 36 face images: 12 target faces (two of each race and sex) and 24 distractor faces containing four faces of each race and sex. Within each race and sex category, a standard face outline template was used, and each target face was created by aligning eyes, nose, and mouth features into the template. Distractor faces for the whole trials were created by replacing one feature (i.e., eyes, nose, or mouth) in the target face with the respective feature of another face of the same race and sex. Part stimuli were created by extracting the eye, nose, or mouth region from each of the target faces and the distractor faces. Target and distractor stimuli for the part trials displayed only the critical feature (see Fig. 3). At a viewing distance of approximately 60 cm, whole faces were of 7.5° horizontal by 10.5° vertical and for isolated features the sizes were: eyes 6.5° × 2.2°; nose 2.6° × 2.2°; mouth 3.8° × 1.9°.

The task comprised three study-test race-blocks (Chinese, Caucasian and African faces). During the study phase, participants were instructed to memorise four faces (two males) and their associated names (e.g., John, James, Jill, and Jane). Each face-name pair was shown for 5 s with an inter-stimulus interval of 1 s. Participants entered the test phase only when they could correctly identify every face-name pair in a single loop; otherwise, an additional reminder would be presented after three iterations. This ensures that participants were familiarised with each face. On each trial in the test phase, a question was presented (e.g. “Which is John’s nose?), followed by a choice of two alternative images presented on the left and right sides of the screen, both horizontally centred. In the part condition, the display consisted of two isolated features (two eyes, two noses, or two mouths), one was from the target face, and the other was from the distractor face. In the whole condition, the display contained two whole faces, with the target and a distractor face differing only with respect to one face part. Participants were required to indicate if the target stimulus was on the left or on the right. The image pair remained on the screen until response.

Stimuli were matched between the two conditions, such that facial parts tested in the part condition were also tested in the whole condition. The whole and part conditions were randomly intermixed. Each block consisted of 24 part and 24 whole trials. The order of block presentation was counterbalanced across participants.

#### Composite task

**Figure 4:**
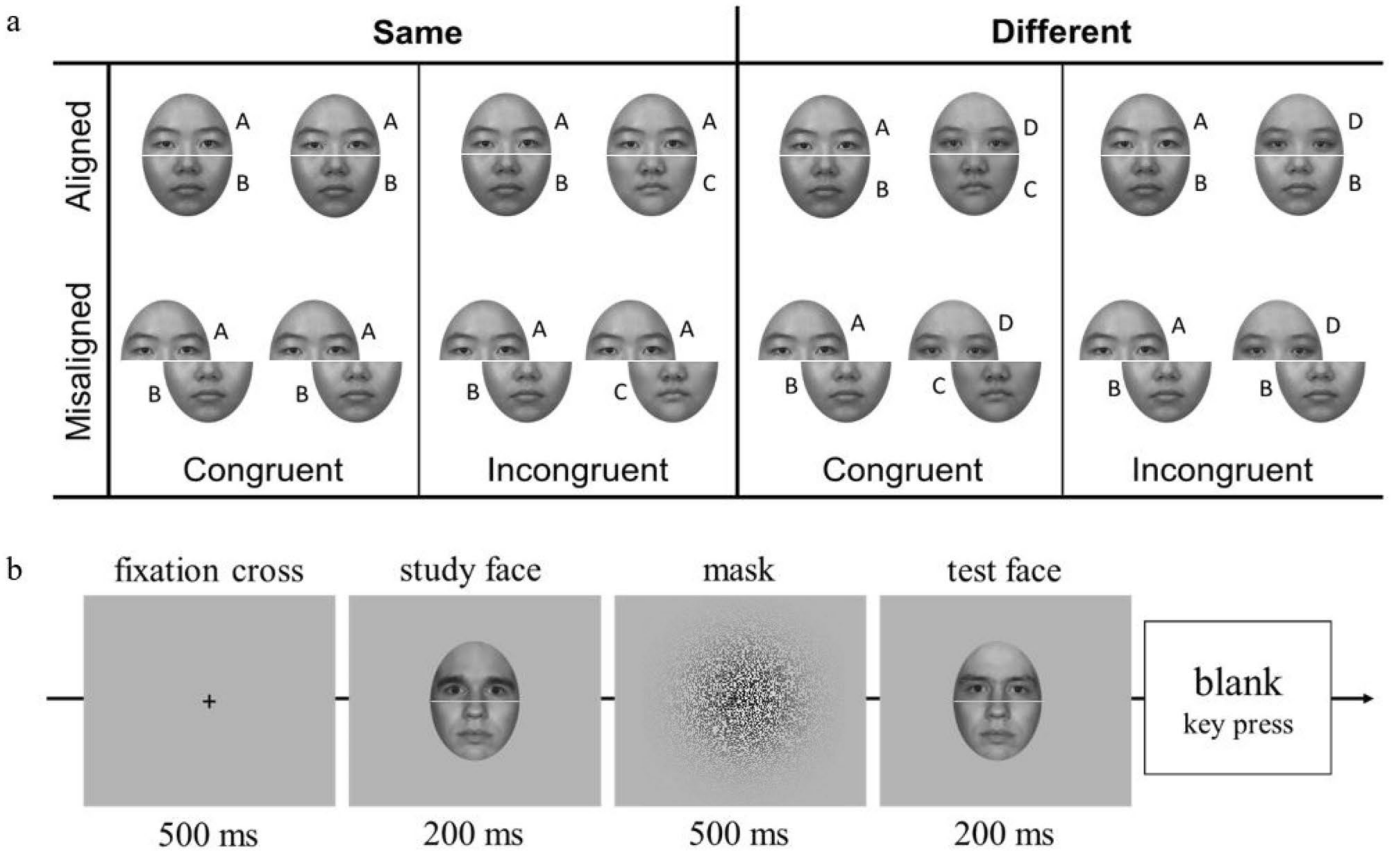
(**a**) Examples of the experimental design and (**b**) a sample of a ‘different’ trial used in composite-face task. The participants’ task was to match the sequentially presented top halves while ignoring the bottom halves.

Faces were generated from 60 images (20 for each race; half females) of Chinese, Caucasian, and African faces. Each face image was divided into two halves horizontally across the middle of the nose using Adobe Photoshop CS6. The top and bottom halves from same-gender faces of different individuals were then recombined at random, leaving a 3-pixel gap between the two parts. The top half and bottom halves were presented either aligned or misaligned (see Fig. 4a). In the misaligned trials, the top and bottom face parts were misaligned by shifting the top half horizontally to the left by half a face width. The same composite faces were used in both conditions. This resulted in 40 aligned and 40 misaligned composite faces in total for each race category. Stimuli in the aligned condition were 7.5° horizontal by 10.5° vertical while stimuli in the misaligned condition were 11. 2° horizontal by 10.5° vertical.

Following Gauthier and Bukach^17^ (Fig. 4a), in congruent trials, the top and bottom parts of the face were created either from the same faces or from different faces (i.e., top-same and bottom-same or top-different and bottom-different). On the other hand, in incongruent trials, one of the face halves was created from the same face, while the other half was created from different faces (i.e., top-same and bottom-different or top-different and bottom-same). This paradigm allows the calculation of a bias-free measure of sensitivity—*d*′ prime^51,52^.

Each trial started with a central fixation cross for 500 ms, followed by a centred face for 200 ms. After a Gaussian noise mask of 500 ms, a test face appeared randomly in one of eight locations, each placed 1.2° from the screen’s centre, for 200 ms. Next, a blank screen was presented until a response was made. The participants’ task was to judge as quickly and accurately as possible whether the top half of the test face was identical to the preceding study face while ignoring the task-irrelevant bottom half. They were instructed to indicate their decision by pressing two keys on a keyboard (see Fig. 4b). On each trial, both faces within a pair were either aligned or misaligned, and these two conditions were intermixed. Trials were blocked by face race, and the order of blocks was counterbalanced across participants. Hence, each participant performed three experimental blocks of 80 trials (40 aligned and 40 misaligned), half of which consisted of face pairs that shared an identical top half (same trials), and half of which consisted of face pairs with different top halves (different trials). Order of trial presentation was fully randomised across participants. Participants first completed 12 practice trials to ensure that they understood the task.

#### Global–local task

**Figure 5:**
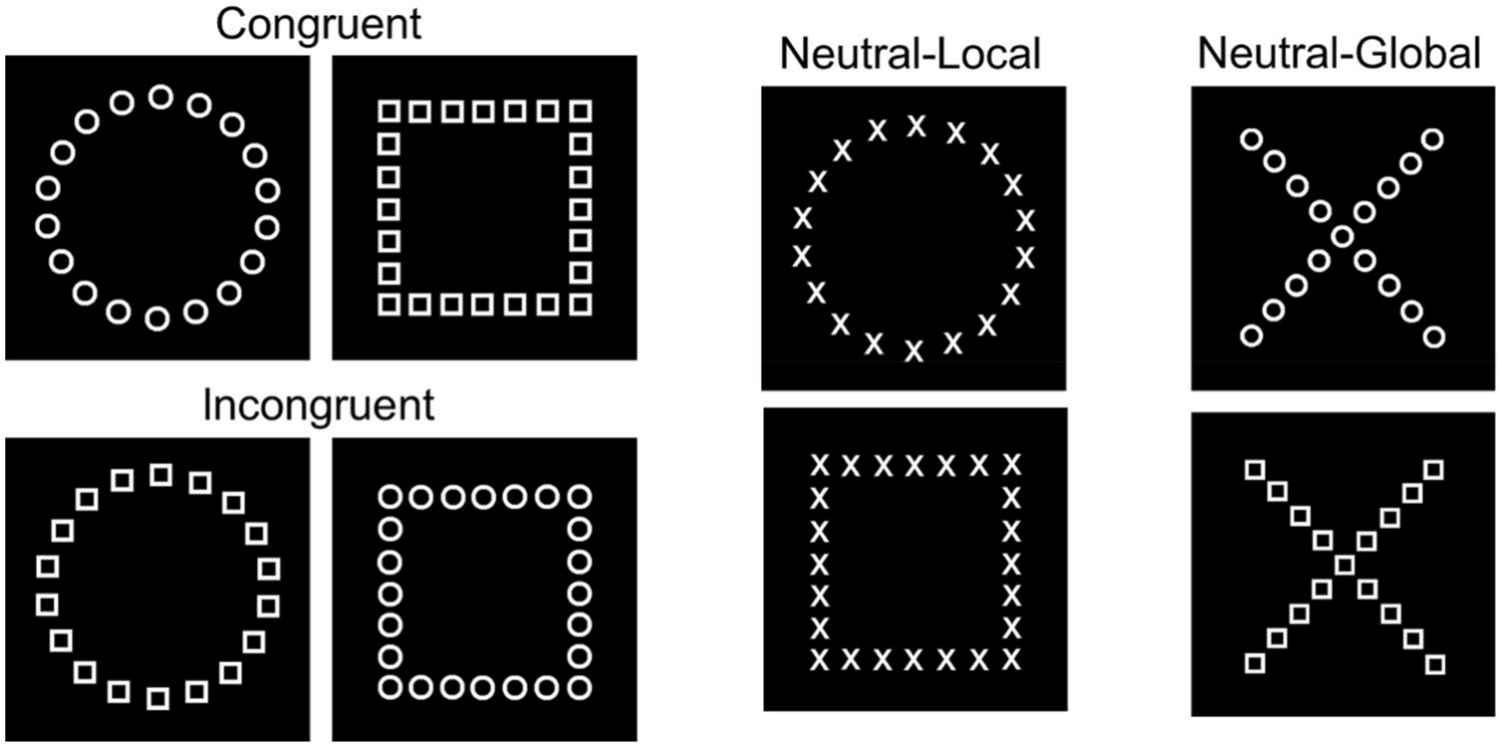
Example of Navon stimuli for global–local task.

This task is a variant of Navon’s^53^ task used in Wang et al.^31^ and assesses participants’ bias to attend to the global shapes versus local shapes, or vice versa. In congruent shapes, the global and the local objects forming the shapes shared an identity (e.g., local squares forming a global square). In incongruent shapes, the shapes at the two levels had different identities (e.g., local circles forming a global square). In addition to congruent and incongruent conditions, we also included a neutral (baseline) condition at both global and local levels in which a task-irrelevant object (an X) forms the global or local shapes (see Fig. 5). The Navon stimuli consisted of shapes (circle, square or cross) with white outline presented on a black background. Each local shape was 0.5° × 0.5°; the local shapes were arranged to form a global square (4.9° × 4.9°), global circle (5.6° × 5.6°), or a global cross (4.9° horizontal × 5.3° vertical).

There were two blocks of trials, each containing 18 practice and 108 test trials. Each block was preceded by instructions to identify the target shapes (circle and square) at either the global or local level as quickly and accurately as possible. In each block, there were 36 congruent trials, 36 incongruent trials and 36 neutral trials (18 local, 18 global). The neutral trials were included to serve as a baseline measure. The three main types of trials were randomly intermixed. Each trial began with a blank screen (500 ms), followed by a central fixation cross (700 ms), Then, a shape stimulus appeared randomly in one of the eight possible locations (0.49° away from the centre of the screen) for 150 ms, followed by a mask (48 × 48 array of diamonds each 0.19° × 0.19°) for 500 ms. Participants were asked to indicate whether the target shape they saw was a circle or a square as fast as possible. This task took approximately 3 min. Each participant completed 216 trials in total (108 local-level and 108 global-level), with 18 practice trials in each block.

## Results

Distributions were normal as indicated by Kolmogorov–Smirnov test (all *ps* ≥ 0.1). The assumptions of homogeneity of variance were met in the three main measures (i.e., *d*′, accuracy, and mean response time) and no violations were detected (Levene’s test all p > 0.05). Prior to each analysis for these three measures, outliers further than two standard deviations from the mean were removed. For each ANOVA, Greenhouse–Geisser corrections were applied whenever sphericity was violated. Follow-up tests were conducted using post-hoc tests with Bonferroni correction for significant main effects and planned comparisons for significant interaction effects. Bonferroni-corrected *p* values were reported. To ensure there was no speed-accuracy trade off, analyses on face task performance were repeated using mean response times (RTs) as the dependent variable. Given that the pattern of results was similar in the accuracy and RT data, in the interest of brevity, we report the response time results in Supplementary Text.

It is frequently argued that support for the null hypothesis being true cannot be obtained from the fact that the p-values are larger than the alpha level (e.g.^54–56^). Thus, in addition to reporting the traditional null hypothesis significance tests, we also performed Bayesian analyses^57,58^ using the statistical software JASP^59^ (0.14.0.0, https://jasp-stats.org/) and the JASP default prior^60,61^ (Cauchy prior, r = 0.707; JASP Team, 2020). Bayesian analysis has the pragmatic benefit that it is not based on the evaluation of significance levels that can be interpreted incorrectly, particularly when the results are non-significant^62^. The Bayes Factor (BF10) provides the likelihood ratio of the probability of the data given the alternative hypothesis (H1) divided by the probability of the same data given the null hypothesis (H0). A BF_10_ value between 1 and 3 provides anecdotal evidence for H_1_; a value between 3 and 10 provides moderate evidence for H_1_; a value above 10 provides strong evidence for H_1_; a value between 1 and 1/3 provides anecdotal evidence for H_0_; a value between 1/10 and 1/3 provides moderate evidence for H_0_ and; a value less than 1/10 provides strong evidence for H_0_.

### Yes–no recognition task

d-prime (*d*′) was used as an index of participants’ face recognition sensitivity. In all cases where hit rate or false alarm rate equals 1.0, Snodgrass and Corwin’s^63^ correction was applied to overcome infinite values of *d*′. The *d*′ scores were then calculated by subtracting each participant’s z-score for false-alarm rates from z-score for hit rates (*d’* = Z_H_ − Z_FA_)^64^. A two-way repeated measure analysis of variance (ANOVA) was performed on *d*′, with face race (Chinese, Caucasian, and African) as within-subjects factor and participant race (Malaysian-Chinese, European-Caucasian, African, and Australian-Caucasian) as between-subjects factor.

#### Recognition accuracy (*d*′)

**Figure 6:**
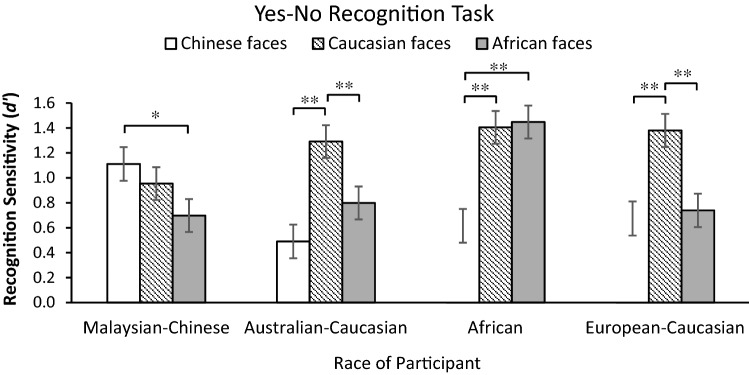
*d’*′ scores for the yes–no face recognition test of own- and other-race faces in Malaysian-Chinese, Australian-Caucasian, African, and European-Caucasian participants. Error bars represent standard errors of the mean (**p < 0.01; *p < 0.05).

Results from the ANOVA revealed a significant main effect of Face Race, *F* (2, 230) = 24.14, *p* < 0.001, *η*_*p*_^2^ = 0.17, BF_10_ = 1.25 × 10^6^, but no main effect of Race of Observer was found, *F* (2, 87) = 1.80, *p* = 0.15, *η*_*p*_^2^ = 0.05, BF_10_ = 0.23. Participants generally had highest recognition performance for Caucasian faces, followed by African faces, and then Chinese faces (all *p*s < 0.05, BF_10_ ≥ 315.58). There was a significant Face Race × Race of Observer interaction, *F* (6, 230) = 8.06, *p* < 0.001, *η*_*p*_^2^ = 0.20, BF_10_ = 2.69 × 10^5^ (see Fig. 6). Pairwise comparisons (with p values Bonferroni corrected for multiple comparisons) confirmed that participants of each of the ethnicities manifested an own-race recognition advantage. Malaysian Chinese were better at recognising own-race faces than African faces (*p* = 0.02, BF_10_ = 15.55), but no difference was found between own-race and Caucasian faces (*p* = 0.95, BF_10_ = 0.25). European-Caucasians showed higher recognition sensitivity towards own-race faces relative to Chinese and African faces (both *p* ≤ 0.001; BF_10_ = 41.75 and BF_10_ = 17.69, respectively). Africans performed better for African and Caucasian faces than for Chinese faces (both *p* < 0.001; BF_10_ = 411.74 and BF_10_ = 1526.83, respectively) while no difference was detected between African and Caucasian faces (*p* = 1, BF_10_ = 0.20). Australian-Caucasians recognised own-race faces better than Chinese (*p* < 0.001, BF_10_ = 312.83) and African faces (*p* = 0.008, BF_10_ = 8.60).

### Whole-part task

The whole part effect (WPE)—an index of holistic face processing—was calcuther-race faces by using the formula lated by subtracting accuracy scores for part trials from those for whole trials. To control for any differences in baseline accuracy, we computed the standardized WPE scores for own- and obelow^65^:

$$WPE=\frac{ (\mathrm{\% }correct \,whole-\mathrm{\% }correct part)}{(\mathrm{\% }correct\, whole+\mathrm{\% }correct \,part)} .$$

#### Whole-part effect (WPE)

**Figure 7:**
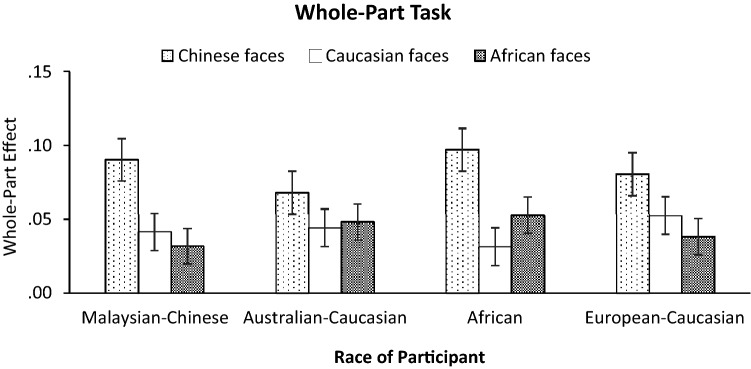
The magnitudes of the whole-part effect (WPE) for own- and other-race faces for each ethnic group in whole-part face task. Error bars indicate standard errors of the mean.

A mixed ANOVA was performed on the magnitude of WPE, with Face Race as within-subjects factor whereas Race of Participant as between-subjects factor. The main effect of Face Race was significant, *F*(2, 234) = 17.61, *p* < 0.001, *η*_*p*_^2^ = 0.13, BF_10_ = 6.29 × 10^4^, such that WPE was stronger for Chinese faces than African (*p* < 0.001, BF_10_ = 4758.91) and Caucasian faces (*p* = 0.002, BF_10_ = 1201.85) while no difference was found between African and Caucasian faces (*p* = 1, BF_10_ = 0.13). Neither the main effect of Race of Participant, *F*(3, 117) = 0.11, *p* = 0.95, *η*_*p*_^2^ = 0.003, BF_10_ = 0.046, nor the critical two-way interaction between Face Race and Race of Participant was significant (see Fig. 7), *F*(5.74, 223.86) = 1.21, *p* = 0.30, *η*_*p*_^2^ = 0.03, BF_10_ = 0.019, suggesting that the magnitude of WPE was not stronger for own-race faces than for other-race faces (Supplementary Table S1). Complementary one-sample *t* tests split by participant race were computed to assess whether the mean WPE scores were significantly positive. Results confirmed that in each race group, the WPE scores were significantly greater than zero, not only for own-race faces, but also for other-race faces (all *ps* < 0.01, BF_10_ ≥ 19.12), indicating the emergence of holistic face processing regardless of the different races of faces.

### Composite face task

Holistic processing in the composite-face task was indicated by the performance differences between the congruent trials and incongruent trials. To further determine whether there was a difference in holistic face processing between own- and other-race faces within each race group, we then computed the composite-face effect (CFE) score for each race of faces separately using the following formula^66^:

$${\text{Congruency effect }} = d^{\prime}\;{\text{congruent trials }}{-}d^{\prime}\;{\text{incongruent trials,}}$$

$${\text{Composite}} - {\text{face effect }}\left( {{\text{CFE}}} \right) \, = {\text{ congruency effect }}\left( {{\text{aligned trials }}{-}{\text{ misaligned trials}}} \right).$$

The magnitude of CFE between race groups was then examined with a mixed ANOVA, involving Face Race as within-subjects variable and Race of Participant as between-subjects variable.

#### Composite face effect (CFE)

**Figure 8:**
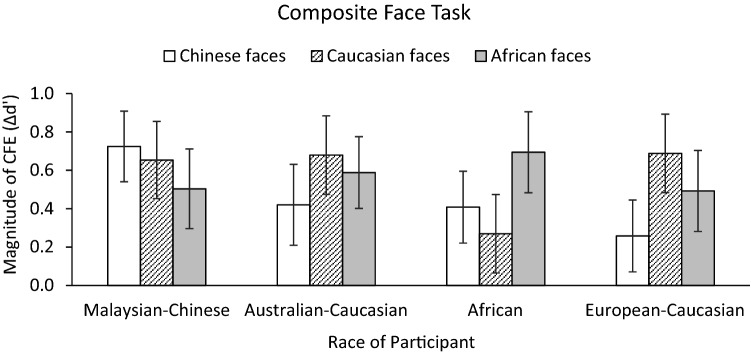
The magnitudes of the composite face effect (CFE) for own- and other-race faces for each ethnic group in composite face task. The CFE was calculated by subtracting congruency effect observed in misaligned condition from that observed in aligned condition (i.e., the alignment by congruency interaction). Error bars indicate ± 1 standard error of the mean.

A 3 (Face Race) by 4 (Race of Participant) ANOVA performed on the *CFE* scores showed that neither the main effect of Race of Participant nor the main effect of Face Race was significant, *F*(3, 117) = 0.44, *p* = 0.72, *η*_*p*_^2^ = 0.01, BF_10_ = 0.027 and *F*(2,234) = 0.14, *p* = 0.87, *η*_*p*_^2^ = 0.001, BF_10_ = 0.034, respectively. No crossover interaction between Race of Participant and Face Race was found (see Fig. 8), *F*(6, 234) = 0.91, *p* = 0.49, *η*_*p*_^2^ = 0.02, BF_10_ = 0.058, indicating similar holistic processing for both own- and other-race faces in each race group (Supplementary Table S2). Complementary one-sample t-tests split by participant race showed that, in most cases, the CFE scores were significantly greater than zero, not only for own-race faces, but also for other-race faces (all *ps* < 0.05, BF_10_ ≥ 2.27 × 10^3^). The only exceptions were the CFEs for Caucasian faces in African participants, *t* (29) = 1.19, *p* = 0.24, and for Chinese faces in European-Caucasian participants, *t* (29) = 1.17, *p* = 0.25.

### Global–local task

Participants’ accuracy was near ceiling across trial types (mostly above 90%). This near-perfect performance could potentially mask the global–local interference effect and render the results less reliable. Therefore, our subsequent analyses focus on the response time (RT) instead to calculate the global–local interference (GLI) scores, as traditionally done (e.g.^31,53,67^). Only RTs for correct responses were included in the analysis and RTs for a trial were discarded if they were shorter than 200 ms or longer than 2000 ms. Preliminary analysis on RTs showed that participants made slowest responses in incongruent trials (*M* = 536 ms), followed by the neutral trials (*M* = 520 ms), and then the congruent trials (M = 503 ms) (all *p* < 0.001), with neutral being faster than congruent trials (*p* = 0.01), suggesting that neutral trials can serve as a baseline measure. Since performance (both accuracy and RT) was not affected by whether the participants were tested on neutral-local (mean accuracy = 93.42%, *SD* = 10.50%; mean RT = 537 ms, *SD* = 57 ms) or neutral-global trials (mean accuracy = 97.02%, *SD* = 4.78%; mean RT = 530 ms, *SD* = 63 ms) (both ps > 0.05), we collapsed across these conditions in the analysis.

To measure participants’ tendency to globally process general objects, a global–local interference (GLI) score was calculated using the following formula for each participant by examining the degree to which global features on the local incongruent trials interfere with RT.

$$GLI= \frac{Congruent\, \left(global-local\right)-Incongruent (global-local)}{Congruent\, \left(global+local\right)+Incongruent (global+local)}.$$

Positive GLI scores indicate a global processing bias whereas negative GLI scores show a local processing bias.

#### GLI

**Figure 9:**
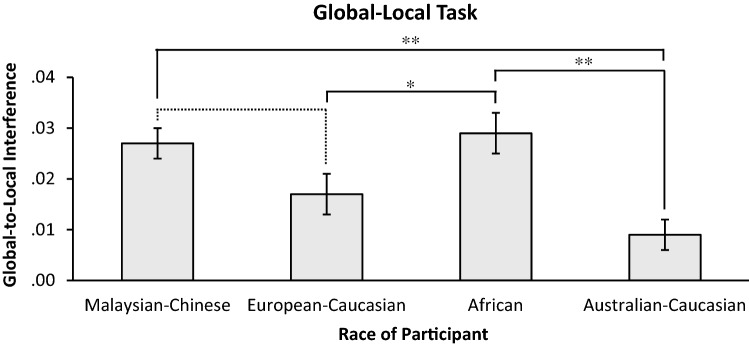
The magnitude of global–local interference (GLI) as a function of participant group. Error bars indicate standard errors of the mean. Asterisks indicate significant differences between race groups (**p < 0.01; *p < 0.05).

As determined by one-way ANOVA, there was a statistically significant difference between race groups (see Fig. 9), *F* (3,117) = 10.81, *η*_*p*_^2^ = 0.22, *p* < 0.001, BF_10_ = 53.32. Pairwise comparisons (with Bonferroni-corrected p values) showed that the magnitude of GLI in Malaysian Chinese were significantly greater than Australian Caucasians (*p* < 0.001, BF_10_ = 19.28) and marginally higher than European Caucasians (*p* = 0.09, BF_10_ = 0.30). Similarly, Africans showed significantly stronger GLI than European Caucasians (*p* = 0.04, BF_10_ = 1.03) and Australian-Caucasians (*p* < 0.001, BF_10_ = 53.70). No significant difference was found between Malaysian Chinese and Africans (*p* = 0.65, BF_10_ = 0.28), or between European- and Australian-Caucasians (*p* = 0.25, BF_10_ = 1.32).

### Correlation analyses

Pearson’s correlation analyses were performed to determine whether the face recognition ability (FRA) for own- versus other-race faces was related to the three holistic processing indices: composite-face effect (CFE), whole-part effect (WPE), and global–local interference (GLI). Rather than completely excluding outliers with many valid observations from the inter-task correlational analyses, cases identified more than 2 SDs from the mean for a particular measure were replaced by a score plus two times the standard deviations. On this basis, less than 2% of the data were replaced within each task (yes–no task: 1.38%; whole-part task: 1.93%; composite-face task: 1.1%; global–local task: 0.83%). After Bonferroni-correct for multiple comparisons, none of the correlations between FRA and measures of holistic processing (Table 1) and between the ORE of FRA and the ORE of holistic processing (Table 2) was statistically significant, suggesting that strength of the ORE in face recognition was not predicted by strength of the ORE in holistic processing. To further support these null findings, we performed the corresponding Bayesian correlation tests (Table 2); for the ease of data visualisation, the scatterplots were created (Supplementary Figs. S3–S6).

**Table 1:** Pearson correlations (and corresponding *p*-values) between the holistic processing indices—whole-part effect (WPE), the composite-face effect (CFE), and global–local interference (GLI)—with face recognition ability (*d*′) as a function of stimulus race in each race group.

	Malaysian Chinese (N = 31)			Africans (N = 30)			Australian-Caucasians (N = 30)			European-Caucasians (N = 30)		
	*chi d*′	*sa d*′	*cau d*′	*chi d*′	*sa d*′	*cau d*′	*chi d*′	*sa d*′	*cau d*′	*chi d*′	*sa d*′	*cau d*′
**WPE**												
Chinese faces	− 0.50 (0.04)	− 0.20 (0.28)	− 0.46 (0.01)	− 0.32 (0.08)	− 0.20 (0.29)	− 0.38 (0.04)	0.15 (0.43)	0.08 (0.69)	− 0.08 (0.69)	− 0.05 (0.81)	− 0.35 (0.06)	− 0.09 (0.63)
African faces	− 0.14 (0.46)	− 0.12 (0.54)	− 0.27 (0.15)	− 0.35 (0.06)	− 0.40 (0.03)	− 0.17 (0.38)	− 0.03 (0.87)	− 0.15 (0.42)	− 0.29 (0.13)	0.01 (0.95)	− 0.26 (0.17)	− 0.01 (0.94)
Caucasian faces	0.28 (0.12)	− 0.01 (0.97)	− 0.02 (0.92)	− 0.06 (0.76)	− 0.02 (0.92)	0.10 (0.60)	− 0.12 (0.92)	− 0.06 (0.74)	0.06 (0.76)	− 0.17 (0.38)	− 0.28 (0.14)	− 0.03 (0.87)
**CFE**												
Chinese faces	0.14 (0.45)	0.24 (0.20)	0.15 (0.41)	0.08 (0.68)	0.30 (0.11)	0.05 (0.80)	0.10 (0.61)	0.03 (0.87)	− 0.08 (0.69)	− 0.22 (0.25)	− 0.05 (0.78)	− 0.23 (0.22)
African faces	0.24 (0.20)	− 0.04 (0.84)	0.15 (0.43)	0.07 (0.71)	− 0.30 (0.11)	− 0.37 (0.04)	− 0.02 (0.90)	0.31 (0.09)	− 0.16 (0.41)	0.15 (0.43)	− 0.13 (0.49)	− 0.16 (0.40)
Caucasian faces	0.31 (0.08)	0.24 (0.20)	0.30 (0.10)	− 0.15 (0.43)	0.11 (0.57)	− 0.20 (0.30)	0.28 (0.14)	0.01 (0.97)	0.06 (0.74)	0.15 (0.42)	0.02 (0.91)	0.04 (0.83)
**GLI**	− 0.02 (0.92)	0.15 (0.43)	0.02 (0.90)	0.34 (0.06)	0.07 (0.70)	− 0.20 (0.28)	0.41 (0.02)	0.18 (0.34)	− 0.02 (0.94)	− 0.15 (0.42)	0.08 (0.69)	− 0.04 (0.82)

*chi* Chinese faces, *sa* South African faces, *cau* Caucasian faces. *α* (two-tailed) = 0.002 (0.05/21).

**Table 2:** Summary of Pearson’s correlations (corresponding *p*-values and Bayes factors) between the ORE of face recognition ability (FRA), the OREs of holistic processing (WPE and CFE), and GLI by the race of observers.

Race of observers	Pearson’s r	*p*	*BF*_10_
**Chinese (N = 31)**			
FRA_chi_cau **− **WPE_chi_cau	− 0.35	0.08	0.93
FRA_chi_cau **− **CFE_chi_cau	− 0.06	0.77	0.23
FRA_chi_sa **− **WPE_chi_sa	− 0.22	0.25	0.66
FRA_chi_sa** − **CFE_chi_sa	− 0.09	0.64	0.48
**European-Caucasian (N = 30)**			
FRA_cau_chi **– **WPE_cau_chi	0.15	0.42	0.31
FRA_cau_chi **– **CFE_cau_chi	− 0.07	0.71	0.24
FRA_cau_sa **– **WPE_cau_sa	0.02	0.93	0.23
FRA_cau_sa **– **CFE_cau_sa	0.06	0.76	0.24
**African (N = 30)**			
FRA_sa_chi **– **WPE_sa_chi	− 0.14	0.47	0.29
FRA_sa_chi **– **CFE_sa_chi	− 0.38	0.12	0.96
FRA_sa_cau **– **WPE_sa_cau	− 0.11	0.56	0.27
FRA_sa_cau **– **CFE_sa_cau	− 0.22	0.25	0.43
**Australian-Caucasian (N = 30)**			
FRA_cau_chi **– **WPE_cau_chi	0.19	0.31	0.37
FRA_cau_chi **– **CFE_cau_chi	− 0.05	0.78	0.24
FRA_cau_sa **– **WPE_cau_sa	0.21	0.27	0.41
FRA_cau_sa **– **CFE_cau_sa	0.30	0.11	0.75

*chi* Chinese faces, *sa* South African faces, *cau* Caucasian faces. α (two-tailed) = 0.01 (0.05/4).

## Discussion

This cross-cultural study aimed to systematically examine the relationship between holistic processing and recognition of own- and other-race faces, by using Malaysian Chinese, African, European-Caucasian, and Australian-Caucasian participants. The current experiment yielded four main results. First, the ORE for recognition performance was pronounced in the face recognition task. Second, participants across race groups did not show stronger holistic processing—as indexed by both the composite-face effect (CFE) and the whole-part effect (WPE)—for own- than other-race faces. Third, in a global–local task, both Malaysian Chinese and African participants were more susceptible to the GLI, indicating a stronger global processing bias as compared to European- and Australian-Caucasian participants. Fourth, the WPE, the CFE, and the GLI were not associated with face recognition performance for other-race faces, indicating that the ORE cannot be accounted for by reduced face processing in global/holistic manner for other-race faces.

Across four race groups, participants exhibited a robust ORE in face recognition memory, although less prominently for Caucasian faces. Most interestingly, Malaysian Chinese participants, who had grown up in a highly multi-ethnic and Western-influenced Asian country, performed equally well at recognising Chinese and Caucasian faces, but less well at recognising African faces. This is consistent with the findings by Wong et al.^48^ and Tan et al.^28^ (but see^27^). The latter study further explained the observed deficit in the recognition of African faces as a product of insufficient visual experience, which leads to a core lack of perceptual ability in the face system to extract the most diagnostic information from that face race. On the other hand, African participants recognised African faces as well as they recognised Caucasian faces but were less good at recognising Chinese faces. In contrast, both European- and Australian-Caucasian participants recognised Caucasian faces better than Chinese and African faces.

Considering the relatively high proportion of ethnic Chinese people in Malaysia (42.3% in the Kuala Lumpur)^68^, we initially anticipated that Africans and European-Caucasian participants, who had resided in the country for half a year or more on average prior to participating in this study, would recognise Chinese faces well. However, this was not the case. The results showed that both African and European-Caucasian exchange/transfer students were generally poor at recognising Chinese faces, indicating that staying in a multiracial environment for a short period of time does not necessarily allow them to develop sensitivity to facial features that are essential for recognising unfamiliar other-race faces. Given the reduced plasticity for face recognition in adulthood^69^, a reduction of ORE would require sufficient individuating experience during childhood^69^ and/or explicit training^70^, rather than mere exposure to other-race faces^71^.

Malaysian Chinese and African participants were able to recognise Caucasian faces equally as well as their own-race faces. These results should not be too surprising, as Malaysian Chinese and African participants, who were students attending a branch campus of a British university, were more likely to have increased exposure to Caucasian faces in the mass media (e.g., western movies). Such a heightened experience in actively individuating them in everyday life might lead to improvements in perceptual sensitivity to diagnostic features on Caucasian faces.

To test the holistic account of the ORE, we used two direct (but uncorrelated^37^) measures of holistic processing: the composite-face and whole-part tasks. In both measures, we did not find evidence of stronger holistic processing effect for own- than other-race faces. This effect is remarkable because it was consistent across all our race groups. Although a few studies have found stronger holistic processing for own compared to other race faces^11,65^, these results are not always replicated. In fact, considerable evidence has accumulated suggesting that holistic processing occurs for other race faces^23,24^, for facial morphologies that are less visually experienced^13,38–40^, and even for other-species faces^72^. Our results thus run counter to the prediction derived from the holistic account of ORE that the magnitude of holistic processing would be stronger for own-race faces than for other-race faces.

It is tempting to interpret our results as showing that the holistic processing for own- and other-race faces is comparable in magnitude. To seek evidence that support the null hypothesis, we additionally performed Bayesian statistical analysis for two lines of results: (a) the magnitudes of holistic processing are not stronger for own- than other-race faces (see Supplementary Table S4); and (b) neither the CFE or WPE are highly correlated with the face recognition performance. The results are summarised in Tables 1 and 2, where the overall pattern of results is consistent with those obtained via NHST (null hypothesis significance testing) analysis. However, one caveat is that, after adjusting for multiple comparisons in the NHST analyses, there were a few cases of a weak, non-significant pattern of stronger holistic effects for own-race or specific-race faces (e.g., there were suggestions of a stronger WPE effect for Chinese and African participants looking at Chinese faces), and so caution should be exercised in drawing this conclusion based on null findings. In addition, despite a very large sample size relative to prior work and a pronounced ORE, in terms of *accuracy*, for the composite-face and whole-part tasks, these measures may not have been sufficiently sensitive to capture racial differences in holistic processing even at standard experimental sizes. Thus, the interpretation of CFE and WPE data must also be taken with caution unless they can be replicated with a larger sample size.

Holistic processing has been found to be associated with face recognition performance^31,73^ and the ORE magnitude^16^. In the present study, however, participants’ memory for own- and other-race faces did not seem to be affected by the magnitude of holistic processing. The failure to find evidence for a correlation is surprising given the dominant theme in the literature that holistic processing is important for both perceiving and recognising faces. This null finding cannot be attributed to any confound derived from the stimulus variability because observers of different races were always better recognising own-race faces (i.e. ORE) across face tasks (see Supplementary Figs. S1, S2).

Rather, it suggests that holistic processing, which lacks reliable individual differences^74^, is not directly associated with differences in recognition memory performance for own- and other-race faces. Extensive individuating experience with own-race faces could enhance face recognition ability^75^, but such experience may not be required to generalise holistic processing to other races of faces. Such an interpretation is consistent with the idea that holistic processing for other-race faces can be easily employed without being restricted by an intrinsic, context-dependent capacity^76^.

Publication bias is a possible explanation when an effect does not replicate^77^. It is relatively easy to publish results showing a difference between two groups, even if the difference was unpredictable, small and hard to explain. It is likely that the published papers overstate the differences in holistic processing between own- and other-race faces. Our current results resonate with several recent studies showing that holistic processing is not directly linked with face recognition ability^18,24^ and can be elicited by both own- and other-race faces without extensive individuating experience^38^. Taken together, these observations challenge the assertion that the ORE in face recognition is a consequence of reduced holistic processing for other-race faces. Holistic processing may play a significant role in the early stages of face recognition^78^, possibly at the level of face detection or face matching that place lower cognitive demands on memory; however, it is not sufficient for explaining the differences in recognition for own- and other-race faces. This rather varied evidence also indicates that the degree of holistic processing applied to a face stimulus may not be as strongly modulated by its perceived race identity as commonly expected; instead, it seemed to be somewhat dependent on the facial physiognomy, stimulus characteristics and tasks performed on them^79,80^.

Overall, our results suggest that, regardless of the race, faces are processed holistically and that there is no strong association between holistic processing and recognition of own and other race faces. These findings have an important theoretical implication, namely that holistic processing is necessary but not sufficient for face identification^81,82^. Although holistic processing would allow the fast binding of facial features into a coherent global percept, this representation would need then to be further processed by a specialised face recognition mechanism^83^. In the same vein, our results support the notion that the origins of holistic face processing are better accounted for by the template hypothesis rather than the attentional strategy hypothesis (for reviews, see^4^). While the attention strategy hypothesis proposes that holistic processing—a strategy of attending to all face parts simultaneously—is shaped by the experience from frequent social interactions and regular exposure to faces^4,19^, the template hypothesis postulates that faces are represented as a single unit to fit a memory template^6,84^ which may be established innately^85^. Our current results that holistic processing can be elicited by both own- and other-race faces without extensive individuating experience seem more consistent with holistic processing being a consequence of the representational constraints of a global face template rather than the inflexibility in attentional weightings on face parts.

Another open question is whether people possess the necessary perceptual abilities to recognise other-race faces at the level of the individual, but only lack the social motivation to do so^86^. According to the social-cognitive position, the source of the ORE is not perceptual, but a resistance to individuate other-race faces due to their out-group status. Hence, the emergence of the ORE may be due to motivational factors rather changes in perceptual expertise. Alternatively, ORE could be a product of converging factors involving social categorization, motivated individuation, and perceptual experience; for example, neither raw perceptual exposure nor the motivation to individuate is sufficient to attenuate the ORE but requires both the proper motivation and practice to individuate other-race faces. Further research is required to confirm these hypotheses.

Here we also provide the first study to use Navon figures to compare global–local processing differences between Malaysian Chinese, African, Australian Caucasian, and European Caucasian participants. Our results show that both Malaysian Chinese and African groups were more susceptible to global–local interference (GLI) than Caucasian groups (European and Australian), indicating a reduced ability to inhibit the influence of holistic information on piecemeal processing. Not only is this result in agreement with numerous studies that provided evidence of stronger global processing in collectivist societies (i.e. the East), and weaker local processing, as compared to individualistic societies (e.g. the West)^41,87^, but also the first report that Africans showed a global processing bias stronger than that of Westerners. This lends strong empirical support to the notion that information-gathering strategy (global versus local processing) for general stimuli can be culture-dependent^25,42^. Furthermore, in line with the domain-specificity hypothesis, the magnitude of GLI did not significantly correlate with holistic face processing measures and face recognition ability, implying that such low-level perceptual biases for information processing may not necessarily be generalizable to high-level face processing tasks.

In conclusion, the current study did not find evidence that holistic processing was stronger for own- than other-race faces. Interestingly, holistic processing for other-race faces did not preclude the observation of OREs. The current findings not only contrast with the assumptions that holistic processing is stronger for own-race faces, but also question the commonly claimed evidence in support of a strong association between face memory and holistic face processing. These results converge with recent studies questioning the holistic processing account of the ORE. Future research is needed to help elucidate the fundamental roles of cognitive and perceptual orienting mechanisms, other than holistic processing, that may underlie the recognition of own- and other-race faces.

## Supplementary Information

Supplementary Information.
