# Supplementary material for: The other-race effect and holistic processing across racial groups
Source: Sci Rep. 2021 Apr 19;11:8507. doi: 10.1038/s41598-021-87933-1 (PMC8055977; doi:10.1038/s41598-021-87933-1)
Supplement: Supplementary file 1 — Supplementary Information. [file 41598_2021_87933_MOESM1_ESM.docx]

The Other-Race Effect and Holistic Processing Across Racial Groups

Hoo Keat Wong^1^, Alejandro J. Estudillo^1, 2^, Ian D. Stephen^3, 4^, & David R.T. Keeble^1^

1 School of Psychology, University of Nottingham Malaysia, Semenyih, Malaysia

2 Department of Psychology, Bournemouth University, Dorset, UK

3 Department of Psychology, Macquarie University, Macquarie Park, Australia

4 Perception in Action Research Centre, Macquarie University, Macquarie Park, Australia

*Correspondence should be addressed to H.K.([hookeat.wong@nottingham.edu.my](mailto:hookeat.wong@nottingham.edu.my))

# Supplementary Tables

Table 1. Mean accuracy scores in percentages *(and standard deviations)* on the whole and part trials (broken down by eyes, nose, and mouth), and the magnitude of whole-part effect (*WPE*) for each race of face split by participant race

|  | Malaysian Chinese (*n=31*) | | | |  | Australian-Caucasian (*n=30*) | | | |  | African (*n=30*) | | | |  | European-Caucasian (*n=30*) | | | |
| --- | --- | --- | --- | --- | --- | --- | --- | --- | --- | --- | --- | --- | --- | --- | --- | --- | --- | --- | --- |
|  | *Eyes* | *Nose* | *Mouth* | *All* |  | *Eyes* | *Nose* | *Mouth* | *All* |  | *Eyes* | *Nose* | *Mouth* | *All* |  | *Eyes* | *Nose* | *Mouth* | *All* |
| Whole trials |  |  |  |  |  |  |  |  |  |  |  |  |  |  |  |  |  |  |  |
| *Chinese faces* | 94.36 (12.81) | 76.21 (14.84) | 91.53 (12.55) | 87.37 *(9.58)* |  | 92.92 (13.17) | 73.75 (18.56) | 75.83 (17.70) | 81.11 (12.59) |  | 89.17 (13.17) | 81.25 (18.56) | 78.75 (17.70) | 83.06 (12.59) |  | 92.92 (13.17) | 71.67 (18.56) | 78.75 (17.70) | 80.83 (12.59) |
| *Caucasian faces* | 91.94 (11.03) | 77.42 (18.86) | 86.69 (11.86) | 85.35 (10.76) |  | 97.92 (11.03) | 90.00 (18.86) | 95.00 (11.86) | 89.31 (10.76) |  | 90.00 (11.02) | 81.67 (18.86) | 92.92 (11.86) | 88.19 (10.76) |  | 95.42 (11.03) | 82.50 (18.86) | 90.00 (11.86) | 94.31 (10.76) |
| *African faces* | 94.36 (13.17) | 78.23 (18.56) | 85.48 (17.70) | 86.02 (12.59) |  | 93.33 (12.81) | 79.17 (14.84) | 90.42 (12.55) | 81.67 *(9.58)* |  | 95.83 (12.81) | 85.00 (14.84) | 92.50 (12.55) | 91.11 *(9.58)* |  | 87.92 (12.81) | 73.75 (14.84) | 83.33 (12.55) | 87.64 *(9.58)* |
|  |  |  |  |  |  |  |  |  |  |  |  |  |  |  |  |  |  |  |  |
| Part trials |  |  |  |  |  |  |  |  |  |  |  |  |  |  |  |  |  |  |  |
| *Chinese faces* | 91.13 (15.37) | 72.98 (19.41) | 83.07 (16.93) | 82.39 (12.14) |  | 90.83 (16.68) | 61.25 (21.14) | 60.42 (21.67) | 69.17 (14.95) |  | 80.42 (16.68) | 59.58 (21.14) | 70.42 (21.67) | 70.14 (14.95) |  | 85.42 (16.68) | 58.33 (21.14) | 63.75 (21.67) | 70.83 (14.95) |
| *Caucasian faces* | 84.27 (14.27) | 69.36 (18.43) | 81.05 (15.32) | 78.23 (10.77) |  | 93.75 (14.27) | 79.17 (18.43) | 86.67 (15.32) | 80.83 (10.77) |  | 87.50 (14.27) | 75.83 (18.43) | 85.00 (15.32) | 82.78 (10.77) |  | 87.08 (14.27) | 75.00 (18.43) | 80.42 (15.32) | 86.53 (10.77) |
| *African faces* | 89.92 (16.68) | 59.27 (21.14) | 69.36 (21.67) | 72.85 (14.95) |  | 90.83 (15.37) | 69.17 (19.41) | 80.00 (16.93) | 75.83 (12.14) |  | 87.50 (15.37) | 75.42 (19.41) | 85.00 (16.93) | 82.64 (12.14) |  | 86.25 (15.37) | 67.92 (19.41) | 73.33 (16.93) | 80.00 (12.14) |
|  |  |  |  |  |  |  |  |  |  |  |  |  |  |  |  |  |  |  |  |
| WPE | |  |  |  |  |  |  |  |  |  |  |  |  |  |  |  |  |  |  |
| *Chinese faces* | 0.03 (0.10) | 0.15 (0.19) | 0.13 (0.17) | 0.10 (0.09) |  | 0.01 (0.10) | 0.09 (0.19) | 0.13 (0.17) | 0.08 (0.09) |  | 0.06 (0.10) | 0.19 (0.19) | 0.06 (0.17) | 0.10 (0.09) |  | 0.05 (0.10) | 0.12 (0.19) | 0.11 (0.17) | 0.09 (0.09) |
| *Caucasian faces* | 0.05 (0.10) | 0.05 (0.16) | 0.03 (0.10) | 0.04 (0.08) |  | 0.02 (0.10) | 0.07 (0.16) | 0.05 (0.10) | 0.05 (0.08) |  | 0.02 (0.10) | 0.04 (0.16) | 0.05 (0.10) | 0.03 (0.08) |  | 0.05 (0.10) | 0.06 (0.16) | 0.07 (0.10) | 0.06 (0.08) |
| *African faces* | 0.02 (0.10) | 0.02 (0.16) | 0.06 (0.12) | 0.03 (0.07) |  | 0.02 (0.10) | 0.09 (0.16) | 0.07 (0.12) | 0.06 (0.07) |  | 0.05 (0.10) | 0.08 (0.16) | 0.05 (0.12) | 0.06 (0.07) |  | 0.01 (0.10) | 0.05 (0.16) | 0.07 (0.12) | 0.04 (0.07) |

Table 2. Mean *d’* scores (*and standard errors*) on the composite-face task as a function of face race, alignment and congruency by race group.

|  | Malaysian Chinese (n=31) | | |  | Australian-Caucasian (n=30) | | |  | African (n=30) | | |  | European-Caucasian (n=30) | | |
| --- | --- | --- | --- | --- | --- | --- | --- | --- | --- | --- | --- | --- | --- | --- | --- |
|  | CHI | CAU | AFR |  | CHI | CAU | AFR |  | CHI | CAU | AFR |  | CHI | CAU | AFR |
| Aligned |  |  |  |  |  |  |  |  |  |  |  |  |  |  |  |
| Congruent | 2.32 | 2.36 | 2.02 |  | 2.06 | 2.49 | 2.28 |  | 1.58 | 1.66 | 1.62 |  | 1.87 | 2.41 | 1.92 |
|  | (0.14) | (0.13) | (0.14) |  | (0.14) | (0.13) | (0.15) |  | (0.14) | (0.13) | (0.15) |  | (0.14) | (0.13) | (0.15) |
|  |  |  |  |  |  |  |  |  |  |  |  |  |  |  |  |
| Incongruent | 1.62 | 1.65 | 1.41 |  | 1.40 | 1.70 | 1.46 |  | 1.05 | 1.28 | 1.15 |  | 1.49 | 1.52 | 1.29 |
|  | (0.12) | (0.13) | (0.13) |  | (0.12) | (0.13) | (0.14) |  | (0.12) | (0.13) | (0.14) |  | (0.12) | (0.13) | (0.14) |
|  |  |  |  |  |  |  |  |  |  |  |  |  |  |  |  |
| Misaligned |  |  |  |  |  |  |  |  |  |  |  |  |  |  |  |
| Congruent | 1.93 | 2.32 | 1.92 |  | 2.02 | 2.32 | 1.88 |  | 1.28 | 1.78 | 1.43 |  | 1.86 | 2.40 | 1.86 |
|  | (0.15) | (0.14) | (0.14) |  | (0.15) | (0.14) | (0.15) |  | (0.15) | (0.14) | (0.15) |  | (0.15) | (0.14) | (0.15) |
|  |  |  |  |  |  |  |  |  |  |  |  |  |  |  |  |
| Incongruent | 1.95 | 2.27 | 1.80 |  | 1.79 | 2.21 | 1.64 |  | 1.45 | 1.66 | 1.36 |  | 1.76 | 2.22 | 1.73 |
|  | (0.13) | (0.14) | (0.13) |  | (0.13) | (0.14) | (0.13) |  | (0.13) | (0.14) | (0.13) |  | (0.13) | (0.14) | (0.13) |
|  |  |  |  |  |  |  |  |  |  |  |  |  |  |  |  |
| CFE *(d’)* | 0.72 | 0.66 | 0.50 |  | 0.43 | 0.68 | 0.59 |  | 0.40 | 0.26 | 0.70 |  | 0.27 | 0.70 | 0.50 |
|  | (0.19) | (0.20) | (0.21) |  | (0.19) | (0.21) | (0.21) |  | (0.19) | (0.21) | (0.21) |  | (0.19) | (0.21) | (0.21) |
|  |  |  |  |  |  |  |  |  |  |  |  |  |  |  |  |

Notes: CHI = Chinese faces, CAU = Caucasian faces, AFR = African faces. Composite-face effect (CFE) = *d’* [(aligned-congruent – aligned-incongruent) – (misaligned-congruent – misaligned incongruent)].

Table 3. Mean response times in *ms (and standard errors)* on the composite-face task as a function of face race, alignment and congruency by race group

|  | Malaysian Chinese (n=31) | | |  | Australian-Caucasian (n=30) | | |  | African (n=30) | | |  | European-Caucasian (n=30) | | |
| --- | --- | --- | --- | --- | --- | --- | --- | --- | --- | --- | --- | --- | --- | --- | --- |
|  | CHI | CAU | AFR |  | CHI | CAU | AFR |  | CHI | CAU | AFR |  | CHI | CAU | AFR |
| Aligned |  |  |  |  |  |  |  |  |  |  |  |  |  |  |  |
| Congruent | 743.99 | 721.76 | 718.72 |  | 678.73 | 650.64 | 662.10 |  | 806.98 | 753.00 | 729.48 |  | 665.98 | 643.06 | 637.30 |
|  | (25.14) | (19.04) | (18.53) |  | (25.14) | (19.04) | (18.53) |  | (25.14) | (19.04) | (18.53) |  | (25.14) | (19.04) | (18.53) |
|  |  |  |  |  |  |  |  |  |  |  |  |  |  |  |  |
| Incongruent | 769.27 | 765.13 | 754.37 |  | 697.54 | 683.16 | 699.15 |  | 822.90 | 798.11 | 741.02 |  | 672.60 | 665.27 | 666.45 |
|  | (22.79) | (23.39) | (20.59) |  | (22.79) | (23.39) | (20.59) |  | (22.79) | (23.39) | (20.59) |  | (22.79) | (23.39) | (20.59) |
|  |  |  |  |  |  |  |  |  |  |  |  |  |  |  |  |
| Misaligned |  |  |  |  |  |  |  |  |  |  |  |  |  |  |  |
| Congruent | 740.12 | 718.03 | 733.25 |  | 682.72 | 646.42 | 660.19 |  | 780.99 | 748.66 | 686.55 |  | 659.75 | 640.82 | 635.75 |
|  | (23.79) | (19.19) | (19.79) |  | (23.79) | (19.19) | (19.79) |  | (23.79) | (19.19) | (19.79) |  | (23.79) | (19.19) | (19.79) |
|  |  |  |  |  |  |  |  |  |  |  |  |  |  |  |  |
| Incongruent | 733.98 | 711.42 | 722.99 |  | 670.27 | 652.44 | 662.36 |  | 770.90 | 754.90 | 702.65 |  | 663.24 | 641.81 | 639.67 |
|  | (24.23) | (19.06) | (19.42) |  | (24.23) | (19.06) | (19.42) |  | (24.23) | (19.06) | (19.42) |  | (24.23) | (19.06) | (19.42) |
|  |  |  |  |  |  |  |  |  |  |  |  |  |  |  |  |
| CFE (*ms*) | 21.07 | 55.82 | 10.86 |  | 16.36 | 32.25 | 12.44 |  | 61.28 | 58.67 | 41.25 |  | 17.95 | 32.59 | 24.58 |
|  | (12.19) | (14.63) | (11.75) |  | (12.19) | (14.63) | (11.75) |  | (12.19) | 14.63 | 11.75 |  | (12.19) | (14.63) | (11.75) |
|  |  |  |  |  |  |  |  |  |  |  |  |  |  |  |  |

Notes: CHI = Chinese faces, CAU = Caucasian faces, AFR = African faces. Composite-face effect (CFE) = *RT* [(aligned-congruent – aligned-incongruent) – (misaligned-congruent – misaligned incongruent)].

Table 4. Multiple paired comparisons between race faces for CFE and WPE by different race of observers, including critical value t, uncorrected *p*-values where *α*=.05, effect sizes (Cohen’s *d*), and Bayes factors.

| Pairwise comparison | | |  | Malaysian Chinese (N=31) | | | |  | European Caucasian (N=30) | | | |  | African (N=30) | | | |  | Australian Caucasian (N=30) | | | |
| --- | --- | --- | --- | --- | --- | --- | --- | --- | --- | --- | --- | --- | --- | --- | --- | --- | --- | --- | --- | --- | --- | --- |
|  |  |  |  | *t* | *p* | *d* | *BF_10_* |  | *t* | *p* | *d* | *BF_10_* |  | *t* | *p* | *d* | *BF_10_* |  | *t* | *p* | *d* | *BF_10_* |
| CFE_chi | - | CFE_cau |  | 0.32 | 0.75 | 0.06 | 0.20 |  | -1.30 | 0.20 | -0.24 | 0.42 |  | 1.50 | 0.14 | 0.27 | 0.53 |  | -1.3 | 0.20 | -0.24 | 0.42 |
| CFE_chi | - | CFE_sa |  | 0.93 | 0.36 | 0.17 | 0.28 |  | -0.56 | 0.58 | -0.10 | 0.23 |  | 1.21 | 0.24 | 0.22 | 0.38 |  | -0.56 | 0.58 | -0.1 | 0.23 |
| CFE_cau | - | CFE_sa |  | 0.67 | 0.51 | 0.12 | 0.24 |  | 0.63 | 0.54 | 0.11 | 0.23 |  | -0.48 | 0.63 | -0.09 | 0.22 |  | 0.63 | 0.54 | 0.11 | 0.23 |
|  |  |  |  |  |  |  |  |  |  |  |  |  |  |  |  |  |  |  |  |  |  |  |
| WPE_chi | - | WPE_cau |  | 2.81 | 0.01 | 0.50 | 5.01 |  | 1.89 | 0.07 | 0.35 | 0.93 |  | 3.07 | 0.005 | 0.56 | 8.72 |  | 1.61 | 0.12 | 0.29 | 0.61 |
| WPE_chi | - | WPE_sa |  | 4.97 | <.001 | 0.89 | 8.24 |  | 2.19 | 0.04 | 0.40 | 1.52 |  | 2.89 | 0.007 | 0.53 | 5.91 |  | 1.10 | 0.28 | 0.20 | 0.34 |
| WPE_cau | - | WPE_sa |  | 0.66 | 0.51 | 0.12 | 0.23 |  | 0.98 | 0.33 | 0.18 | 0.30 |  | -1.32 | 0.20 | -0.24 | 0.43 |  | -0.31 | 0.76 | -0.06 | 0.20 |

Note: chi = Chinese faces, sa = South African faces, cau = Caucasian faces.

# Supplementary Figures

**

**

**

**

*

*

*Figure 1.* d-prime in the composite task for Chinese, Caucasian and African faces in Malaysian Chinese, Australian-Caucasian, African, and European-Caucasian participants. Error bars represent standard errors of the mean. (***p*<.01; **p*<.05).

**

**

**

**

**

**

**

*

*Figure 2.* Mean percentages of correct responses as a function of stimulus race and participant race. Error bars represent standard error. (***p*<.01; **p*<.05).


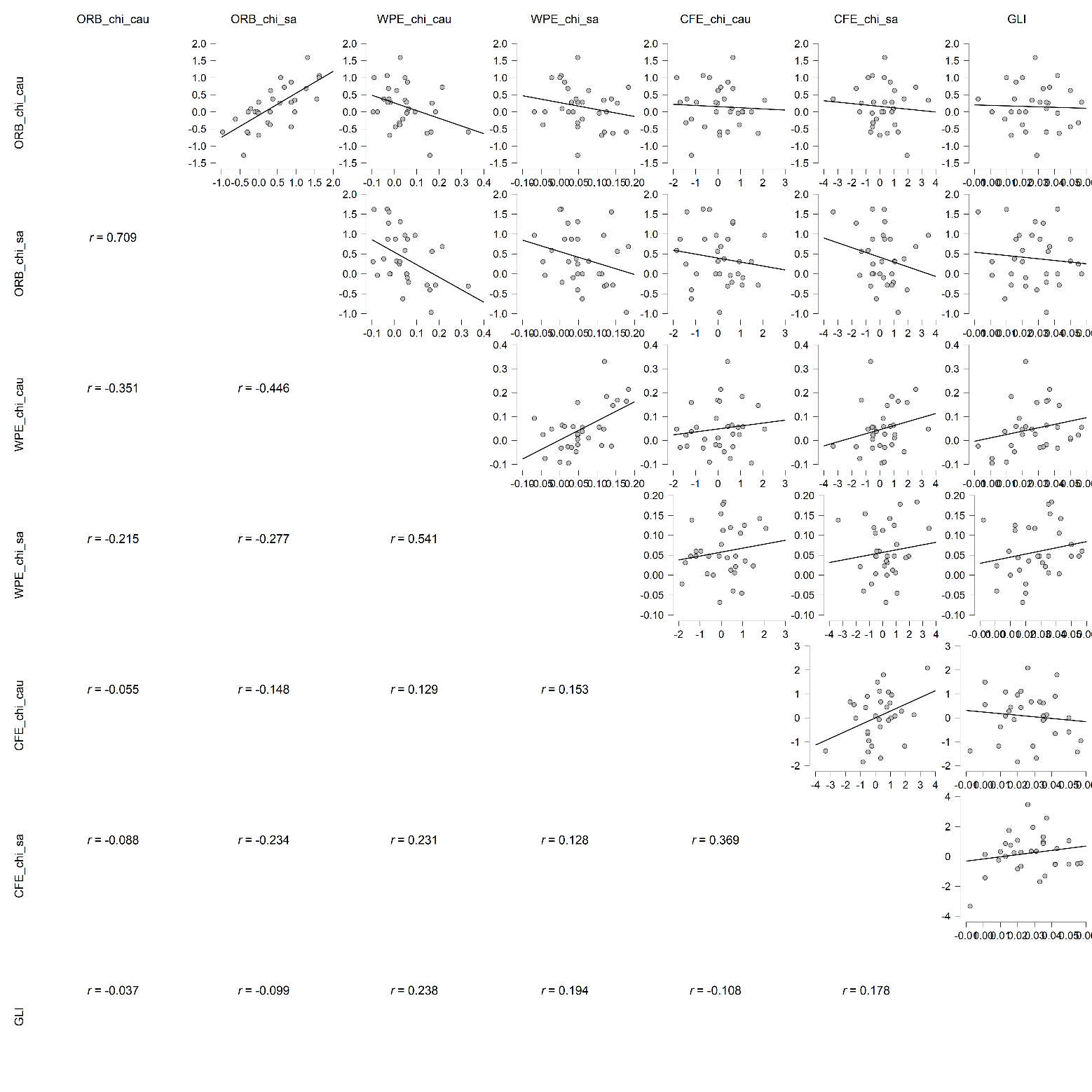


*Figure 3.* Scatterplots of relationships between the ORE in face recognition ability, the OREs of holistic processing indices, and the GLI in Malaysian Chinese participants.


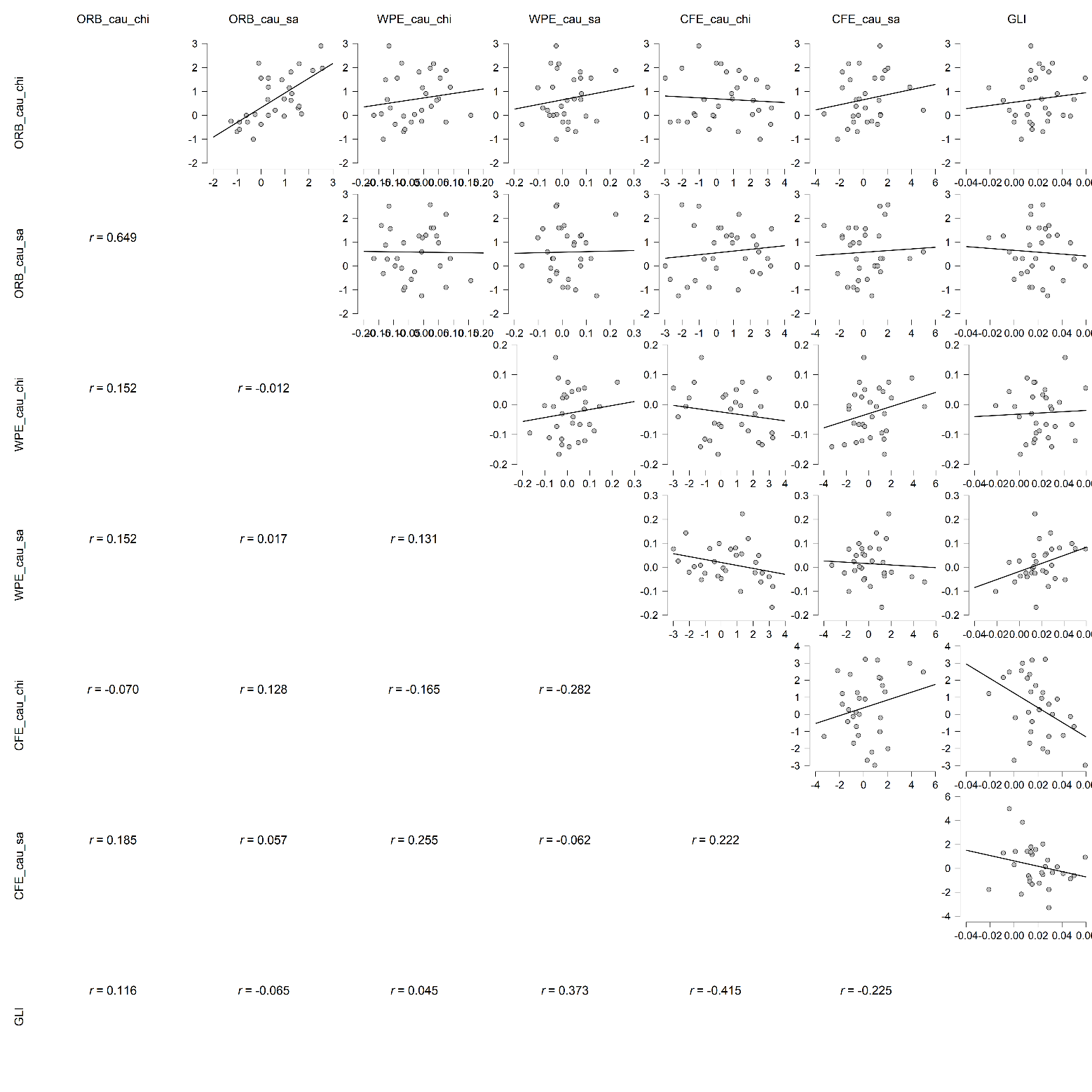


*Figure 4.* Scatterplots of relationships between the ORE in face recognition ability, the OREs of holistic processing indices, and the GLI in European-Caucasian participants.


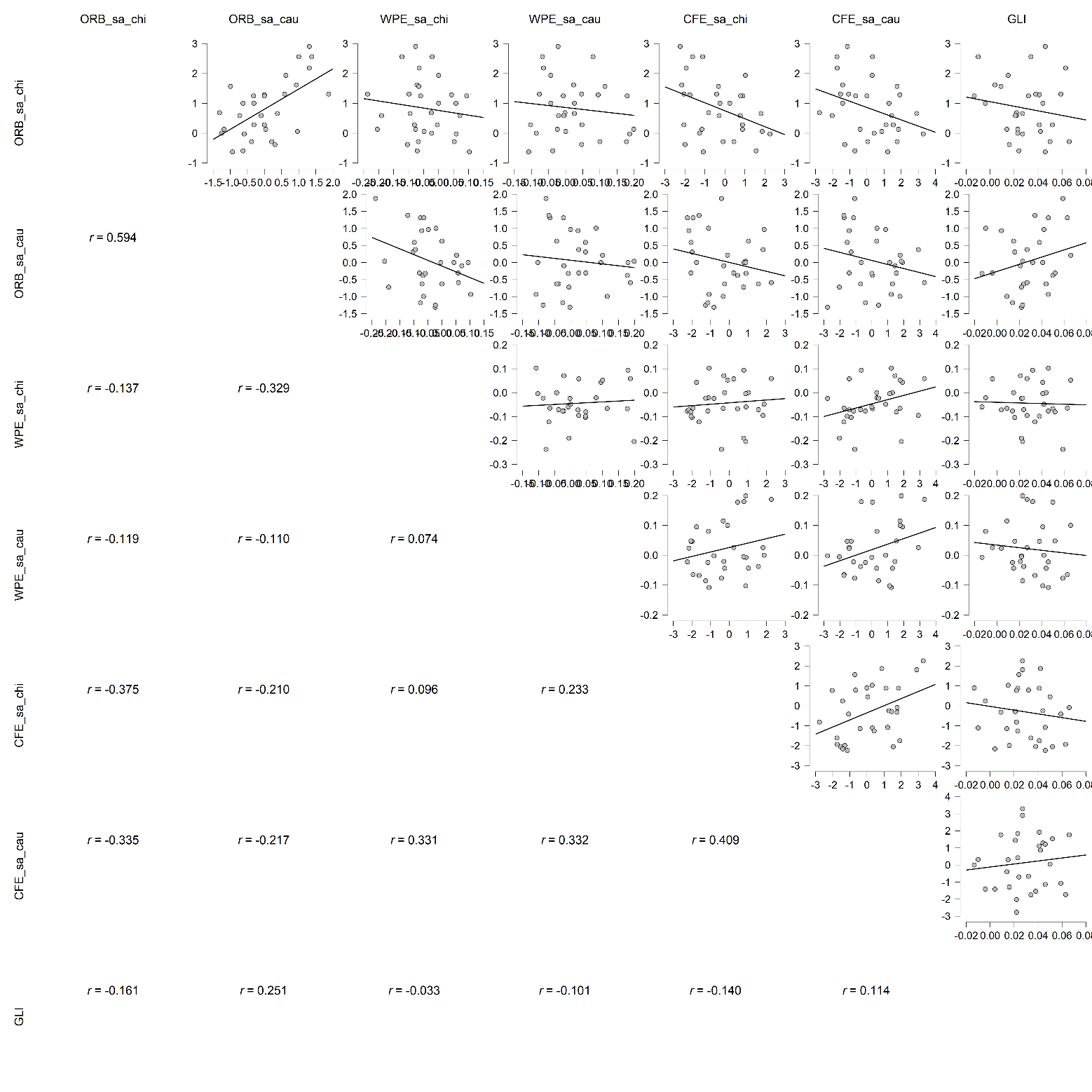


*Figure 5.* Scatterplots of relationships between the ORE in face recognition ability, the OREs of holistic processing indices, and the GLI in African participants.


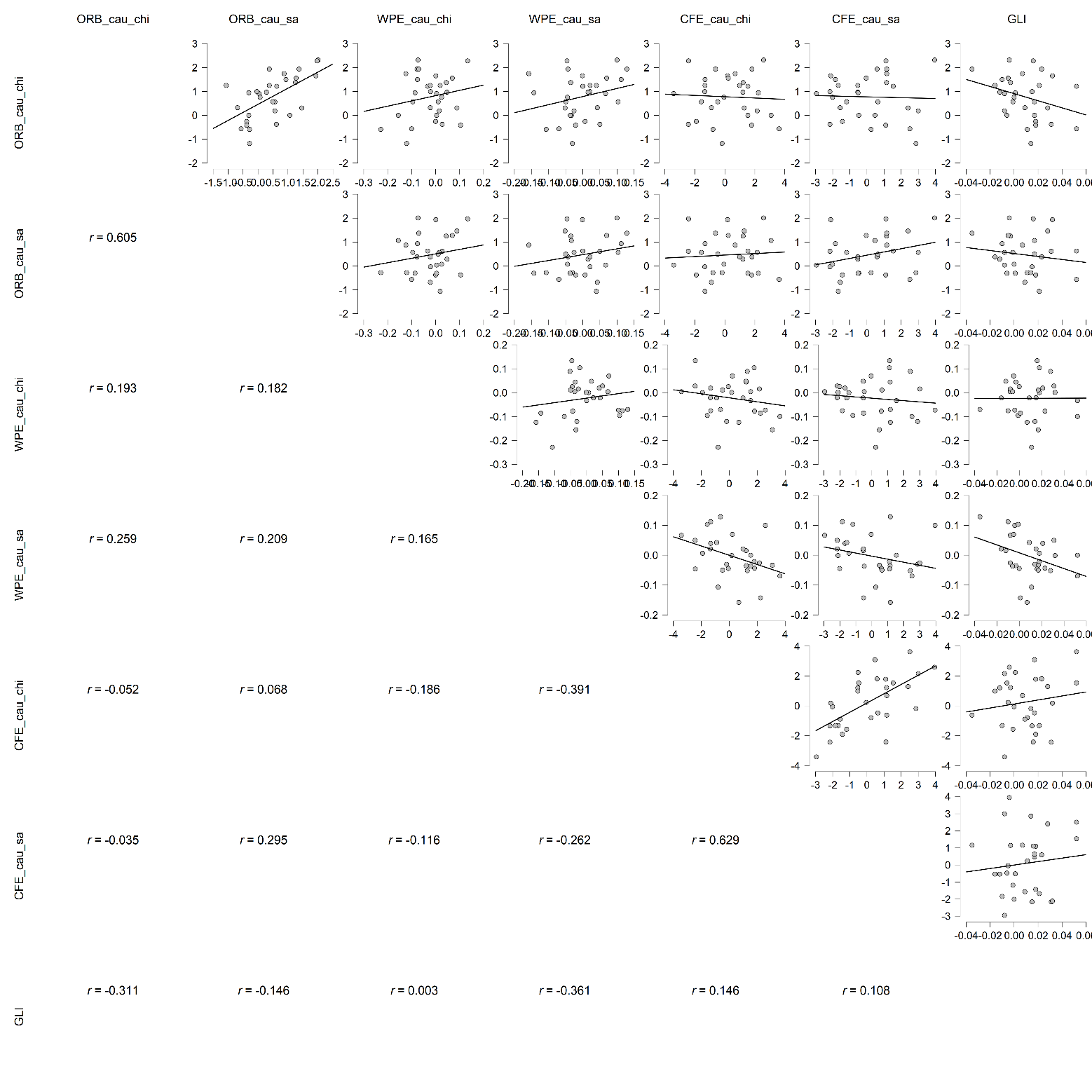


*Figure 6.* Scatterplots of relationships between the ORE in face recognition ability, the OREs of holistic processing indices, and the GLI in Australian-Caucasian participants.

**Supplementary Text**

**Yes-no recognition task**

The main effect of Face Race was significant, *F* (2, 234) = 9.29, *p*<.001, *η_p_^2^* = .07, showing that Caucasian faces (*M*=1.41s) received shorter response times compared to Chinese (*M*=1.52s) (*p*=.003) and African faces (*M*=1.53s) (*p*<.001). There was also a significant main effect of Race of Participant, *F* (3, 117) =7.15, *p*=.002, *η_p_^2^* =.12, such that African participants (*M*=1.78s) made slower responses than Chinese (M=1.43s) (*p*=.04) and Australian-Caucasian (*M*=1.28s) (p=.002) participants did, and marginally slower than European-Caucasians did (*M*=1.45s) (p=.08). However, the interaction between Face Race and Race of Observer interaction was not significant, *F* (6,234) = 0.21, *p*=.97.

**Composite face task**

An analysis of mean correct response times confirmed that differences in accuracy scores could not be attributed to speed-accuracy trade-offs. Descriptive statistics for each experimental condition by race groups are reported in Supplementary Table 3. An ANOVA revealed significant main effects of Alignment, *F* (1,112)=35.63, *p*<.001, *η_p_^2^* = .23, and Congruency, *F*(1,112)= 15.00, *p*<.001, η_p_^2^ = .11, accompanied by a significant Alignment × Congruency interaction, *F* (1,112)=30.96, *p*<.001, *η_p_^2^* = .21. Participants generally responded more quickly on congruent (*M*=701ms) than incongruent (*M*=728 ms) trials (*p*<.001) when faces were aligned, but not when faces were misaligned (*p*=.40). A significant main effect of Face Race was also found, *F* (2, 224) = 4.06, *p*=.02, *η_p_^2^* = .03. Participants tended to make slower responses to Chinese faces (*M*=726ms) than African faces (*M*=699ms) (*p*=.05), while there was no difference between Chinese and Caucasian faces (*M*=709ms) (*p*=.19), or between African and Caucasian faces (*p*=.71). There was also a main effect of Race of Participant, *F* (3, 112) =4.38, *p*=.006, *η_p_^2^* = .10. Both European-Caucasian (*M*=672ms) and Australian-Caucasian (*M*=678ms) participants responded significantly faster than Chinese (*M*=750ms) and African participants did (*M*=747ms) (all *p*< .05), while the response time did not differ between Chinese and African participants (*p*=1). A significant Face Race × Race of Participant interaction was also found, *F* (6,224) =2.41, *p*=.05, *η_p_^2^* = .05, with African participants being considerably faster for own-race faces than for Chinese (*p*=.004) and Caucasian faces (*p*=.01). No other main effects and interactions were significant (*p*>.05).

**Whole-part task**

A mixed factorial ANOVA revealed significant main effects of Face Race, *F*(2, 234)=11.23, *p*<.001, *η_p_^2^* = .09, and Race of Participant, *F*(3,117)=12.08, *p*<.001, η_p_^2^ = .25, on mean response time (RT). The main effect of Face Race was driven by a significantly longer RT made in trials composed of Chinese faces (*M*=3.52s) relative to trials composed of either Caucasian (*M*=3.24s) (p<.001) or African faces (*M*=3.34s) (p=.01). The main effect of Race of Participant was driven by a significantly longer RT made by African participants (*M*=4.08) compared with Chinese (*M*=3.35s), European-Caucasian (*M*=3.00s), and Australian-Caucasian participants (*M*= 3.04s) (all *p*≤.001). However, the interaction between Face Race and Race of Participant failed to reach statistical significance, *F*(6,234)=1.55, *p*=.16, suggesting that participants did not respond significantly faster to own-race faces than to other-race faces. There was also a main effect of Feature, F(1.89, 209.57)=37.22, *p*<.001, *η_p_^2^*=.25, Greenhouse-Geisser corrected. Participants generally made faster responses on eye trials (*M*=3.15s), followed by mouth (*M*=3.38s) and nose trials (*M*=3.57s) (all *p* <.001).

The main effects mentioned above were qualified by a significant three-way interaction involving Face Race, Race of Participant, and Feature, *F* (12, 444) = 2.57, *p*=.003, *η_p_^2^* =.07. Bonferroni-corrected post hoc tests revealed that Chinese participants had tendencies to respond more slowly to the mouth of Chinese faces than that of African and Caucasian faces (both *p*=.06). African participants made slower responses to Chinese-eyes than Caucasian- and African-eyes (both *p*=.01), but no significant difference was found for the nose and mouth trials (all *p*>.05). European-Caucasian participants spent less time on Caucasian mouths than Chinese mouths (*p*=.05), and more time on Chinese eyes than Caucasian (*p*=.03) and African eyes (*p*=.01). Australian-Caucasian participants spent significantly less time on the eyes of Caucasian faces than those of Chinese and African faces (*p*=.001 and *p*=.003, respectively), and less time responding to Caucasian mouths than to Chinese mouths (*p*<.001). Moreover, they showed a tendency to respond faster to African mouths than to Chinese mouths (*p*=.06). Taken together, this indicates that the effect of face parts on RT depends on the combination of participant and stimulus race.

In addition, there was a significant main effect of Condition, *F* (1, 117)=233.66, *p*<.001, *η_p_^2^* =.68, with longer RTs in the whole condition(*M*=3.66s) than in the part condition (*M*=3.07s). The two-way interactions between Feature and Condition, *F*(2, 234) = 28.11, *p*<.001, *η_p_^2^* = .20 , and between Face Race and Condition, *F*(2,234) = 9.16, p<.001, *η_p_^2^* = .08, were significant, accompanied by a three-way interaction, *F*(3.72, 435.09)=2.11, *p*=.04, *η_p_^2^* = .02, Greenhouse-Geisser corrected. To investigate this interaction in more detail, multiple pairwise comparisons (Bonferroni-corrected) between Feature and Condition were carried out within each stimulus race. It was found that, for Chinese faces, participants generally responded faster to the eyes than to the nose and mouth (both *p*<.001) particularly in the whole condition, while no difference between features was detected in the part condition (all *p*>.05). For Caucasian faces, regardless of the whole-part conditions, participants spent more time on the nose trials than the eye and mouth trials (all *p*≤.001). Also, their responses towards the eyes were faster than the mouth when those face parts were presented in the context of whole faces (*p*=.001) rather than when presented alone (*p*=1). For African faces, participants made the fastest responses to the eyes, followed by the mouth and then the nose (all *p*≤.001) in the whole condition; however, no significant difference between face parts was observed in the part condition (all *p*>.05).
